# Activation of Human NK Cells by *Bordetella pertussis* Requires Inflammasome Activation in Macrophages

**DOI:** 10.3389/fimmu.2019.02030

**Published:** 2019-08-27

**Authors:** Michiel M. Kroes, Rob Mariman, Daniëlle Hijdra, Hendrik-Jan Hamstra, Karlijn J. W. M. van Boxtel, Jos P. M. van Putten, Jelle de Wit, Elena Pinelli

**Affiliations:** ^1^Center for Immunology of Infectious Diseases and Vaccines, Center for Infectious Disease Control, National Institute for Public Health and the Environment, Bilthoven, Netherlands; ^2^Department of Infectious Diseases and Immunology, Faculty of Veterinary Medicine, Utrecht University, Utrecht, Netherlands

**Keywords:** inflammasome, NLRP3, crosstalk, interferon-gamma, interleukin-18, innate immunity, *Bordetella pertussis*, human

## Abstract

Pertussis is a highly contagious respiratory infection caused by the bacterium *Bordetella pertussis*. Humans are the only known natural reservoir of *B. pertussis*. In mice, macrophages and NK cells have a key role in confining *B. pertussis* to the respiratory tract. However, the mechanisms underlying this process, particularly during human infections, remain unclear. Here we characterized the activation of human macrophages and NK cells in response to *B. pertussis* and unraveled the role of inflammasomes in this process. NLRP3 inflammasome activation by *B. pertussis* in human macrophage-like THP-1 cells and primary monocyte-derived macrophages (mo-MΦ) was shown by the visualization of ASC-speck formation, pyroptosis, and the secretion of caspase-mediated IL-1β and IL-18. In contrast to macrophages, stimulation of human CD56^+^CD3^−^ NK cells by *B. pertussis* alone did not result in activation of these cells. However, co-culture of *B. pertussis*-stimulated mo-MΦ and autologous NK cells resulted in high amounts of IFNγ secretion and an increased frequency of IL-2Rα^+^ and HLA-DR^+^ NK cells, indicating NK cell activation. This activation was significantly reduced upon inhibition of inflammasome activity or blocking of IL-18 in the mo-MΦ/NK cell co-culture. Furthermore, we observed increased secretion of proinflammatory cytokines in the *B. pertussis*-stimulated mo-MΦ/NK co-culture compared to the mo-MΦ single culture. Our results demonstrate that *B. pertussis* induces inflammasome activation in human macrophages and that the IL-18 produced by these cells is required for the activation of human NK cells, which in turn enhances the pro-inflammatory response to this pathogen. Our data provides a better understanding of the underlying mechanisms involved in the induction of innate immune responses against *B. pertussis*. These findings contribute to the knowledge required for the development of improved intervention strategies to control this highly contagious disease.

## Introduction

Pertussis, also known as whooping cough, is a highly contagious and acute disease of the upper respiratory tract, which can be fatal in newborns and non-vaccinated young children. Pertussis is caused by the Gram-negative bacterium *Bordetella pertussis* (1, 2) and humans are the only known natural reservoir for this pathogen (3). Despite pertussis being a vaccine preventable disease, it has reemerged in vaccinated populations (4, 5). Proposed reasons for this reemergence include pathogen adaptation and waning of vaccine-induced immunity (6–8). Prevention and control of this disease requires new and improved intervention strategies for which a better understanding of the underlying mechanisms involved in shaping a protective immune response is crucial.

The innate immune system is the first line of defense against invading microorganisms. Upon activation, it immediately combats microbes and additionally orchestrates an adaptive immune response. Innate immune cells, including dendritic cells (DCs) and macrophages, contribute to *B. pertussis* induced immunity (9–11). Sensing of *B. pertussis* by murine DCs and macrophages has been shown to result in inflammasome activation (9, 12). Inflammasomes are multiprotein complexes that form in the cytosol of immune cells, particularly in macrophages (13, 14). The best characterized inflammasomes are composed of a specific sensor protein of the nucleotide-binding oligomerization domain-like receptor (NLR) family, the apoptosis-associated speck-like protein containing a caspase activation and recruitment domain (ASC) adaptor protein and pro-caspase-1 (15). Activation of the sensor protein results in the formation of a single, compact speck by the ASC protein, which is essential for the oligomerization and activation of caspase-1 (16). Active caspase-1 cleaves pro-IL-1β and pro-IL-18 resulting in the release of bioactive IL-1β and IL-18, and induces pyroptosis, a form of proinflammatory cell death (17–19). In mice, inflammasome activation is associated with the induction of an antigen-specific T helper (Th) 17 response and clearance of the pathogen (9, 12). Whether *B. pertussis* induces inflammasome activation in human cells and whether this enhances the immune responses against this pathogen is unknown.

Another innate immune cell that has been shown to be essential in the clearance of *B. pertussis* is the natural killer (NK) cell. In mice lacking NK cells, *B. pertussis* disseminates from the respiratory tract and causes a lethal infection (20, 21). One of the main functions of NK cells during bacterial infections is the secretion of the proinflammatory cytokine IFNγ (22). Disruption of IFNγ signaling during a murine infection with *B. pertussis* results in a lethal disseminating disease (21). Furthermore, IFNγ enhances the killing of *B. pertussis* by murine macrophages (23). These studies imply an essential role for IFNγ secreting NK cells in the protective immune response against *B. pertussis* in the mouse model. However, the mechanism by which NK cells are activated during *B. pertussis* infection and how the secretion of IFNγ is induced is unknown in mice and humans.

Since inflammasome activation in macrophages results in the secretion of IL-18 and this cytokine is known to activate NK cells (24–27), we investigate the potential crosstalk between human macrophages and NK cells in response to *B. pertussis* and the role of inflammasomes in this process. We show for the first time that *B. pertussis* induces inflammasome activation in human macrophages and that caspase-mediated IL-18 release is required for the activation of NK cells by the pathogen.

## Materials and Methods

### Ethics Statement

This study was conducted according to the principles described in the Declaration of Helsinki. Buffy coats were provided by the Sanquin Blood Supply. For the collection of samples and subsequent analyses, all blood donors provided written informed consent. Blood samples were processed anonymously and the research goal, primary cell isolation, required no review by an accredited Medical Research Ethics Committee, as determined by the Dutch Central Committee on Research involving human subjects.

### Culture Media

THP-1 cells (InvivoGen) were cultured in Roswell Park Memorial Institute 1640 medium (RPMI; Gibco) enriched with 10% fetal bovine serum (FBS; Gibco), 100 U/ml penicillin, 100 μg/ml streptomycin, 29.2 μg/ml L-Glutamine (Gibco), and 100 μg/ml Normocin^TM^ (InvivoGen), from here on referred to as RPMI culture medium. NK cells were cultured in Iscove's Modified Dulbecco's Medium (IMDM) supplemented with 10% FBS, 100 U/ml penicillin, 100 μg/ml streptomycin, and 29.2 μg/ml L-Glutamine, from here on referred to as IMDM culture medium. Monocytes were differentiated to macrophages in IMDM supplemented with 1% FBS, 100 U/ml penicillin, 100 μg/ml streptomycin, 29.2 μg/ml L-Glutamine, and 50 U/ml human GM-CSF (PreproTech), from here on referred to as monocyte differentiation medium. mo-MΦ and mo-MΦ/NK co-cultures were stimulated in IMDM medium enriched with 1% FBS and 29.2 μg/ml L-Glutamine (Lonza), from here on referred to as infection medium. HEK-Blue IL-1R and HEK-Blue-Null1 cells were cultured in Dulbecco's Modified Eagle Medium (DMEM; Gibco) enriched with 10% FBS, 100 U/ml penicillin, 100 μg/ml streptomycin, 100 μg/ml Normocin^TM^, and 29.2 μg/ml L-Glutamine, from here on referred to as HEK-Blue culture medium.

### Bacterial Strains and Growth Conditions

The streptomycin and nalidixic acid resistant *B. pertussis* Tohama I derivative, B0213, and a *B. pertussis* clinical isolate from 2015, B4393, were used in this study. To ensure consistency in bacterial inoculates between experiments, flash freeze vials (FFV) of both strains were prepared. To prepare the FFV, the bacteria were plated on Bordet Gengou (BG) agar plates, supplemented with 1% glycerol and 15% defibrinated sheep blood (BD Bioscience) and incubated at 35°C and 5% CO_2_ for 4 days. Next, bacteria were sequentially passaged on two successive days on BG agar plates and incubated at 35°C and 5% CO_2_ for 1 day. Bacteria were collected from the BG agar plates and suspended and extensively washed in Thalen-IJssel medium (28). Bacterial suspensions were prepared at OD_590_ 0.5 in Thalen-IJssel medium supplemented with 15% glycerol and snap frozen prior to storing at −80°C. After thawing, FFV were spun down for 10 min at 16,000 × g and the pellet was suspended in infection medium prior to use for cellular *in vitro* infection. To ensure that the freezing process did not affect bacterial viability, colony forming units were confirmed on BG agar plates.

### THP-1 Cell Culture

The THP-1, THP-1 ASC-GFP, and THP-1 NLRP3 deficient cell lines (InvivoGen) were cultured in RPMI culture medium at 37°C and 5% CO_2_. Every other passage selective antibiotics, 100 μg/ml Zeocin^TM^ (InvivoGen) for THP-1 ASC-GFP and 200 μg/ml Hygromycin B Gold (InvivoGen) for NLRP3 deficient THP-1, were added to the RPMI culture medium.

### Human Monocyte and NK Cell Isolation and Macrophage Differentiation

Buffy coats from healthy human donors were used for the isolation of CD56^+^CD3^−^ NK cells and CD14^+^ monocytes for the subsequent generation of mo-MΦ. First, peripheral blood mononuclear cells (PBMC) were obtained by gradient centrifugation of buffy coats diluted eight times in PBS at 1,000 × g for 30 min on Lymphoprep (Nycomed). PBMC were either frozen at −80°C in 50% IMDM culture medium, 10% dimethylsulfoxide (DMSO; Sigma), and 40% FBS for later isolation of NK cells or were used for monocyte isolation using magnetically activated cell sorting in combination with anti-CD14 microbeads (Miltenyi Biotec). The purity of the CD14 positive cells was determined by flow cytometry. For this, monocytes were stained with anti-CD14-PE (BD Biosciences) followed by data acquisition on the LSRFortessa X-20 (BD Biosciences). Data analysis was performed using FlowJo software (version 10.5.3; Tree Star). Monocyte purity was more than 90% for every donor. To facilitate differentiation to mo-MΦ, monocytes were cultured in flat-bottom 96-well culture plates (Greiner) at 150,000 cells/well in monocyte differentiation medium, at 37°C and 5% CO_2_ for 6 days. Monocyte differentiation medium was refreshed after 3 days. NK cells were isolated from the frozen PBMC fraction using the NK Cell Isolation Kit human (Miltenyi Biotec). The purity of the NK cells was determined by flow cytometry. NK cells were stained with anti-CD56-PE/Cy7 (BioLegend) and anti-CD3-BUV395 (BD Biosciences) followed by data acquisition on the LSRFortessa X-20 (BD Biosciences). Data analysis was performed using the FlowJo software (Tree Star). NK cell (CD56^+^CD3^−^) purity was more than 90% for every donor.

### THP-1 Cell Stimulation

THP-1 cells were seeded into flat-bottom 96-well plates at 50,000 cells/well in RPMI culture medium containing 50 ng/ml Phorbol 12-myristate 13-acetate (InvivoGen) to induce differentiation toward a macrophage (MΦ)-like phenotype. After a 22 h incubation at 37°C and 5% CO_2_, cells were washed and incubated in RPMI culture medium for 3 additional days. MΦ-like THP-1 cells were incubated with the indicated amounts of Tohama I. As a positive control for NLRP3 inflammasome activation MΦ-like THP-1 cells were primed for 3 h with 100 ng/ml LPS-EK (InvivoGen) and stimulated with 5 μg/ml Nigericin (InvivoGen) for an additional 22 h. When indicated, MΦ-like THP-1 cells were stimulated in the presence of 10 μg/ml of the caspase inhibitor, Z-VAD-FMK (InvivoGen). Stimulations were performed at 37°C and 5% CO_2_ for 22 h after which supernatant was collected for further analysis.

### Mo-MΦ Stimulation

Mo-MΦ were stimulated with Tohama I or the clinical strain B4393 at a multiplicity of infection (MOI) of 10 in IMDM infection medium. When indicated, mo-MΦ were incubated with 20 μg/ml caspase inhibitor for 30 min and subsequently stimulated with *B. pertussis* in the presence of 10 μg/ml caspase inhibitor. Stimulations were performed at 37°C and 5% CO_2_ for 22 h after which supernatant was collected for cytokine detection.

### NK Cell Stimulation

NK cells were seeded into round-bottom 96-wells plates (Greiner) at 150,000 cells/well in IMDM culture medium supplemented with 5 ng/ml recombinant human IL-15 (rhIL-15; PeproTech) (29). Next, NK cells were immediately stimulated with *B. pertussis* B4393 at a MOI of 10 in the presence or absence of 5 ng/ml recombinant human IL-18 (rhIL-18; R&D systems), 10 ng/ml rhIL-6 (Miltenyi Biotec), 10 ng/ml rhTNFα (PeproTech), or 10 ng/ml rhIL-1β (InvivoGen). Stimulations were performed at 37°C and 5% CO_2_ for 18 h after which BD GolgiPlug^TM^ containing Brefeldin A (BD Biosciences) was added to the culture for 4 h, to inhibit cytokine secretion, before collecting the NK cells for flow cytometry analysis.

### Mo-MΦ/NK Co-culture

NK cells were rested for 22 h in IMDM culture medium supplemented with 5 ng/ml rhIL-15 and incubated at 37°C and 5% CO_2_ to ensure maturation of the NK cells (29). Mature NK cells were added to autologous mo-MΦ in a 1:1 ratio. Co-cultures were stimulated with *B. pertussis* B4393 at a MOI of 10. When indicated, mo-MΦ were incubated with 20 μg/ml caspase inhibitor, 2 μg/ml anti human IL-18 (αhIL-18; InvivoGen), or 2 μg/ml of the isotype control human IgA2 (hIgA2; InvivoGen) for 30 min prior to addition of NK cells in a 1:1 ratio and stimulation of the co-culture. Stimulations were performed at 37°C and 5% CO_2_ for 22 h after which supernatants were collected for cytokine detection and NK cells were collected by washing the wells with PBS for FACS analysis.

### Cytokine and LDH Release Analysis

IL-1β and IL-6 were measured in the supernatant of THP-1 cell cultures by using a Ready-SET-Go ELISA kit (eBioscience) according to the manufacturer's instructions. Immulon 2 HB flat-bottom 96-well plates (Thermo Fisher Scientific) were used for all ELISAs. Mature biologically active IL-1β was measured in the supernatant of THP-1 cell cultures by using the HEK-Blue IL-1β receptor (IL1R) cell line (InvivoGen). The HEK-Blue IL1R cell line and the parental HEK-Blue Null1 cell line contain an NF-κB-inducible secreted embryonic alkaline phosphate (SEAP) reporter gene. IL-1β signaling via the IL1 receptor on the HEK-Blue IL1R leads to expression of SEAP, which activity can be detected in the culture supernatants after adding the substrate Quanti-Blue (InvivoGen). To control for endogenous NF-κB activation, the parental HEK-Blue-Null1 cells (InvivoGen) were used. HEK-Blue IL1R and HEK-Blue-Null1 cells were seeded into flat-bottom 96-wells plates at 50,000 cells/well in HEK-Blue culture medium containing 10% supernatant derived from THP-1 cell cultures or 2 ng/ml rhIL-1β (InvivoGen), serving as a positive control. After a 22 h incubation at 37°C, supernatants were collected and the Quanti-Blue substrate was added. After 2 h of incubation with the substrate, the OD values, indicating SEAP activity, were measured at 639 nm. Measurements and data analysis were performed with a BioTek PowerWave 340, using Gen5 software (version 1.11; BioTek). The concentrations of the cytokines IL-1β, IL-18, IL-23, GM-CSF, IL-10, TNFα, IFNγ, and Granzyme B in the supernatant from mo-MΦ and NK single- and co-cultures were determined using a ProcartaPlex Mix & Match luminex kit (Invitrogen) according to the manufacturer's instructions. Measurements and data analysis were performed with the Bio-Plex 200, using Bio-Plex Manager software (version 6.1; Bio-Rad Laboratories). LDH release was determined as an indicative of pyroptosis by using the CytoTox 96 Non-Radioactive Cytotoxicity Assay Kit (Promega) according to the manufacturer's instructions.

### Inflammasome qPCR Array

mRNA levels of 84 genes associated with inflammasomes were quantified using the RT^2^ Profiler^TM^ PCR Array Human Inflammasomes (QIAGEN) according to the manufacturer's instructions. In short, mo-MΦ were stimulated with *B. pertussis* at a MOI of 100 for 6 h after which the cells were lysed using QIAzol lysis reagents (QIAGEN) and stored at −80°C prior to RNA isolation using the RNeasy mini kit (QIAGEN) following the manufacturer's protocol. cDNA was synthesized from 200 ng total RNA using the RT^2^ First Strand kit (QIAGEN) following the manufacturer's protocol. cDNA was used together with the RT^2^ SYBR Green qPCR Mastermix (QIAGEN) in the RT^2^ Profiler^TM^ PCR Human Inflammasomes Array (QIAGEN) according to the manufacturer's instructions. Data was acquired on a StepOnePlus Real-Time PCR System (Applied Biosystems). Relative transcription levels were analyzed using the web-based software available on www.qiagen.com.

### Flow Cytometry Analysis

NK cells were stained with anti-CD56 PE/Cy7, anti-CD16 BV510, anti-CD25 BV421, anti-HLA-DR BV650, anti-CD69 APC (all from BioLegend), Fixable Viability Stain 780 (BD Biosciences), and anti-CD3 BUV395 (BD Biosciences) for 30 min at 4°C, followed by washing in FACS buffer [PBS pH 7.2; 0.5% BSA (Sigma); 2 mM EDTA (Merck)] and fixed with 2% paraformaldehyde (Merck). For the detection of NK cell-expressed cytokines, NK cells were permeabilized and fixed using the Fixation/Permeabilization Solution kit (BD Biosciences) following the manufacturer's protocol and stained with anti-IFNγ AF700 (BioLegend). NK cells were analyzed for marker expression on the LSRFortessa X-20 (BD Biosciences) and analyzed using FlowJo software (Tree Star).

### Flow Imaging

Mo-MΦ stimulated with the clinical *B. pertussis* strain (B4393) at MOI 100 for 22 h were washed with PBS and incubated with StemPro^TM^ Accutase^TM^ Cell Dissociation Reagent (Thermo Fisher Scientific) to detach the cells. Detached mo-MΦ were washed with FACS buffer and permeabilized and fixed using the Fixation/Permeabilization Solution kit (BD Biosciences) following the manufacturer's protocol. Permeabilized mo-MΦ were stained with the ASC-specific antibody anti-TMS1 (Abcam) for 40 min at 4°C. After extensive washing with PermWash (BD Biosciences) mo-MΦ were stained with the secondary antibody Goat anti-Rabbit IgG H&L (Alexa Fluor 488) (Abcam) for 40 min at 4°C. ASC-speck formation was imaged using the ImageStream MARK II (Merck). Data was analyzed using the Image Data Exploration and Analysis software (Merck).

### Statistical Analysis

Statistical significance was calculated using GraphPad Prism software (version 7). A Wilcoxon matched-pairs signed rank test followed by a bonferroni correction was used. A *p*-value of <0.05 was considered statistically significant.

## Results

### *B. pertussis* Induces NLRP3 Inflammasome Activation in Human Macrophage-Like THP-1 Cells

**Figure 1:**
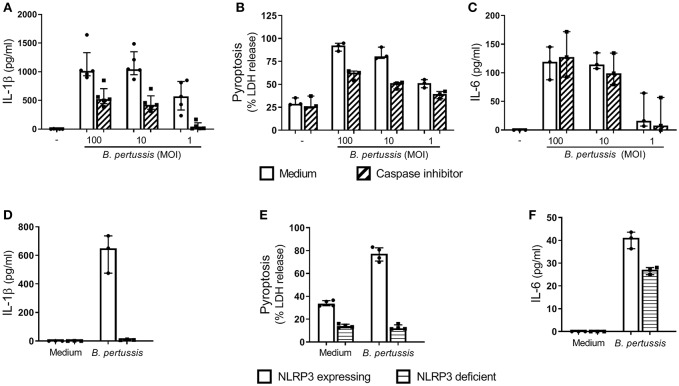
*B. pertussis* induces NLRP3 inflammasome activation in human MΦ-like THP-1 cells. MΦ-like THP-1 cells were stimulated with *B. pertussis* (Tohama I, MOI = 100, 10 or 1) or left untreated for 22 h in the presence (dashed bars) or absence (clear bars) of the caspase inhibitor, Z-VAD-FMK. **(A)** IL-1β (*n* = 5) and **(C)** IL-6 (*n* = 3) were measured in the supernatant of at least three independent experiments. **(B)** LDH release (*n* = 3) was determined with a Cytotoxicity Assay. LDH release is shown as a percentage of the LDH released relative to the percentage of LDH released in the positive control, LPS + nigericin (100% cell death), for NLRP3 activation. **(D–F)** MΦ-like NLRP3 deficient THP-1 cells (horizontal lines) were incubated with *B. pertussis* (Tohama I, MOI = 10) for 22 h. **(D)** IL-1β (*n* = 3) and (**F**) IL-6 (*n* = 3) levels were measured in the supernatant using ELISAs. **(E)** The LDH released by MΦ-like NLRP3 deficient THP-1 cells (*n* = 4) was shown as relative to the LDH release from fully lysed cultures. Results are expressed as medians with interquartile range from at least three independent experiments. Black dots represent the average values from each experiments.

In order to determine whether *B. pertussis* can induce inflammasome activation in human macrophages, we first used the human cell line THP-1, which was differentiated toward a MΦ-like phenotype. Stimulation of these MΦ-like THP-1 cells with *B. pertussis* (Tohama I) for 22 h resulted in a robust dose-dependent IL-1β secretion (Figure 1A). This response was inhibited in the presence of a caspase inhibitor (Z-VAD-FMK) (Figure 1A). Complete inhibition of IL-1β secretion by the caspase inhibitor was observed when the MΦ-like THP-1 cells were stimulated with the lowest bacterial concentration (MOI 1). These findings indicate that IL-1β secretion induced by *B. pertussis* was mediated by inflammasome activation. Using an IL-1β bioassay we confirmed that the mature form of IL-1β was secreted by *B. pertussis-*stimulated MΦ-like THP-1 cells (Figure S1). Another hallmark of inflammasome activation is the induction of a form of programmed cell death, named pyroptosis. When the MΦ-like THP-1 cells were stimulated with *B. pertussis*, a caspase-mediated increase in the release of LDH, a measure for pyroptosis, by these cells was observed (Figure 1B). The caspase inhibitor did not have an effect on caspase-independent activation of MΦ-like THP-1 cells by *B. pertussis*, as indicated by the unaffected secretion of IL-6 by these cells (Figure 1C).

To determine whether *B. pertussis* activates the NLRP3 inflammasome, NLRP3 deficient THP-1 cells were stimulated with *B. pertussis* (Tohama I) at a MOI 10. This yielded very low levels of IL-1β (<10 pg/ml) compared to the NLRP3 expressing THP-1 cell line (Figure 1D). Additionally, *B. pertussis* induced 76.8% cell death of the NLRP3 expressing MΦ-like THP-1 cells, indicated by LDH release, whereas only 12.8% cell death of the NLRP3 deficient MΦ-like THP-1 cells was observed (Figure 1E). Although the *B. pertussis*-stimulated NLRP3 deficient THP-1 cells showed a strong reduction in LDH release and abrogation of IL-1β secretion, these cells were still capable of secreting the inflammasome-independent pro-inflammatory cytokine IL-6 in response to this pathogen (Figure 1F). These findings show that the *B. pertussis*-induced IL-1β secretion and cell death of MΦ-like THP-1 cells is dependent on the NLRP3 inflammasome.

### *B. pertussis* Induces Inflammasome Activation in Primary Human Monocyte-Derived Macrophages

**Figure 2:**
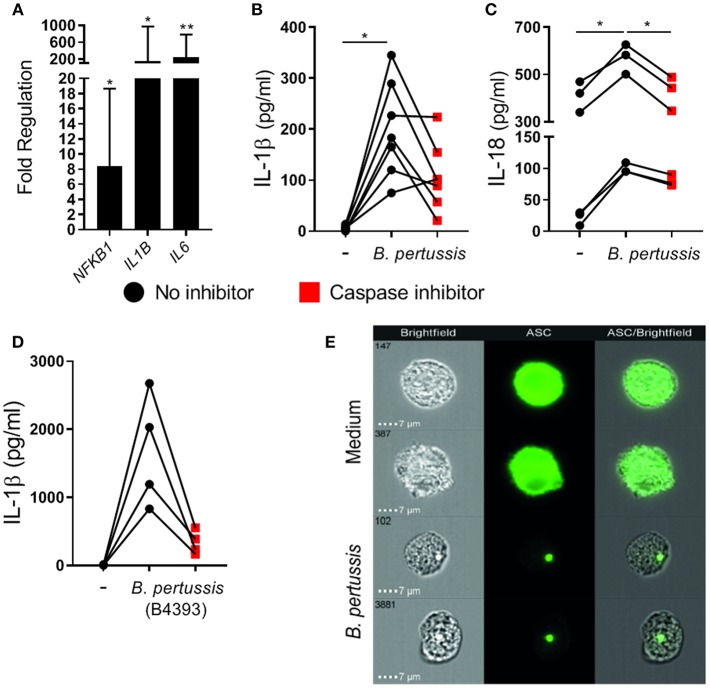
*B. pertussis* induces inflammasome activation in primary human mo-MΦ. **(A)** Mo-MΦ were stimulated with *B. pertussis* (Tohama I, MOI = 100) for 6 h after which the transcription levels of inflammasome associated genes were determined using reverse transcriptase qPCR. Data is expressed as mean fold change of three donors calculated as the transcription levels relative to the transcription levels in untreated mo-MΦ. **(B)** The levels of IL-1β (*n* = 7) and **(C)** IL-18 (*n* = 6) released into the supernatant by mo-MΦ stimulated with *B. pertussis* for 22 h in the presence (red squares) or absence (black dots) of a caspase inhibitor (Tohama I, MOI = 10). **(D)** IL-1β secretion of mo-MΦ stimulated with a clinical *B. pertussis* strain (B4393, MOI = 10) in de presence (red squares) or absence (black dots) of a caspase inhibitor. Black dots and red squares represent values of individual donors. **(E)** Representative images of the cellular ASC (green) distribution as determined by flow imaging of untreated mo-MΦ or mo-MΦ stimulated with a clinical *B. pertussis* strain (B4393, MOI = 100). ^*^p < 0.05, ^**^p < 0.01.

To determine whether *B. pertussis* could also induce inflammasome activation in primary human mo-MΦ, fully differentiated mo-MΦ were stimulated with *B. pertussis* (Tohama I) at a MOI of 100. We first measured the transcription levels of 84 inflammasome-associated genes in mo-MΦ from three different donors after 6 h stimulation with *B. pertussis* relative to untreated cells, using reverse transcriptase qPCR (Figure S2). In the *B. pertussis-*stimulated mo-MΦ a significant increase in the mRNA levels of, among others, transcription factor *NFKB1, IL1B*, and *IL6* was observed (Figure 2A). We then determined whether *B. pertussis* could induce IL-1β and IL-18 release by human primary cells by stimulating the mo-MΦ with *B. pertussis* (Tohama I) at a MOI 10. In the supernatant of *B. pertussis-*stimulated mo-MΦ, significantly increased levels of IL-1β (Figure 2B) and IL-18 (Figure 2C) were detected as compared to untreated mo-MΦ. In the presence of the caspase inhibitor, the secretion of both cytokines was reduced (Figures 2B,C).

To determine whether a recently circulating strain of *B. pertussis* was also able to induce inflammasome activation, human mo-MΦ were stimulated with the clinical *B. pertussis* isolate B4393. Figure 2D shows that this clinical *B. pertussis* isolate induces high levels of IL-1β which were inhibited in the presence of the caspase inhibitor, indicating inflammasome-dependent IL-1β secretion. Additionally, inflammasome assembly in mo-MΦ upon *B. pertussis* (B4393) stimulation was visualized by the formation of the ASC-speck as shown by flow cytometry imaging (Figure 2E). In the medium control, ASC is evenly distributed throughout the mo-MΦ as indicated by the completely green fluorescent cells, whereas, in the *B. pertussis*-stimulated mo-MΦ a compact ASC-speck is observed. Further experiments were performed using this clinical *B. pertussis* isolate.

All together, these data indicate that *B. pertussis* is able to induce inflammasome activation in primary human mo-MΦ.

### IL-18 Primes Human NK Cells to Produce IFNγ in Response to *B. pertussis*

**Figure 3:**
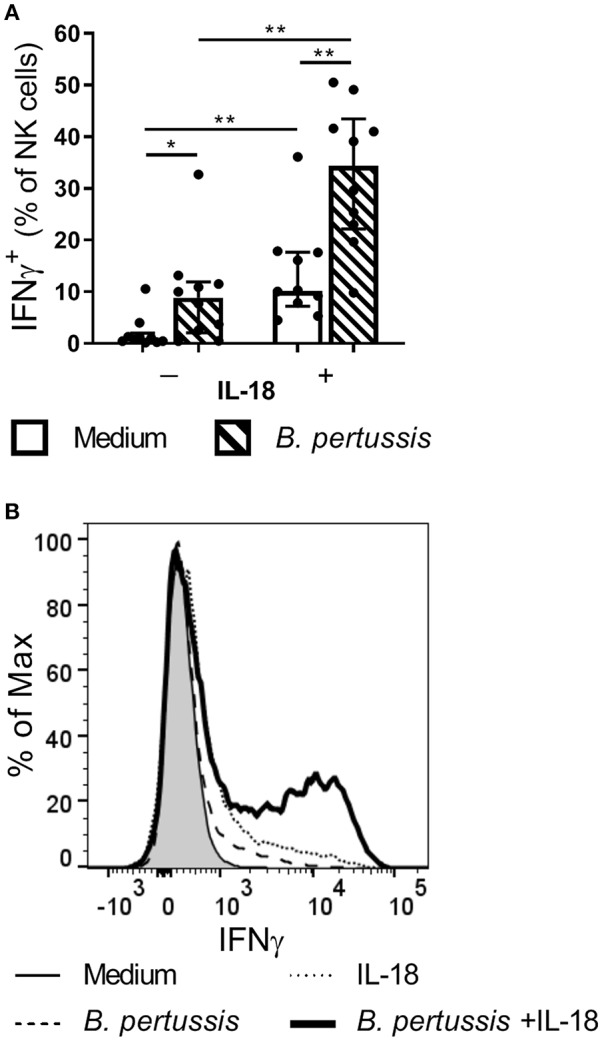
IL-18 primes NK cells to produce IFNγ in response to *B. pertussis*. CD56^+^CD3^−^ NK cells were incubated with medium (clear bars) or *B. pertussis* (B4393, MOI = 10, dashed bars) in the presence or absence of 5 ng/ml rhIL-18 for 18 h after which Brefeldin A was added for 4 h to inhibit cytokine secretion. **(A)** Stimulated NK cells were intracellularly stained for IFNγ and the percentage of IFNγ^+^CD56^+^CD3^−^ NK cells was analyzed using flow cytometry (*n* = 10). Results are expressed as medians with interquartile range. Black dots represent values of individual donors. **(B)** Results are expressed as a histogram of IFNγ^+^CD56^+^CD3^−^ NK cells from one representative donor. ^*^p < 0.05, ^**^p < 0.01.

Since NK cells have been shown to play a critical role in the clearance of *B. pertussis* from the murine respiratory tract, we aim to unravel mechanisms by which human NK cells are activated by the pathogen. Primary human NK cells, isolated from 10 different donors, stimulated with *B. pertussis* (B4393) showed some IFNγ production (Figure 3). Stimulation of NK cells with the known activator IL-18 (24–27), resulted in an average of 10.2% IFNγ^+^ NK cells (*p* = 0.004). Interestingly, stimulation of NK cells with *B. pertussis* in the presence of rhIL-18 yielded a synergistic increase from 8.87 to 34.35% IFNγ^+^ NK cells compared to stimulation in the absence of rhIL-18 (*p* = 0.004) (Figure 3). When stimulating NK cells in the presence of other inflammatory cytokines namely, IL-6, TNFα or IL-1β with or without *B. pertussis*, no significant increase in IFNγ production was observed (Figure S3). These data show that *B. pertussis* enhances IFNγ secretion by IL-18-activated NK cells.

### Increased Proinflammatory Cytokine Secretion in *B. pertussis*-Stimulated mo-MΦ/NK Co-cultures

**Figure 4:**
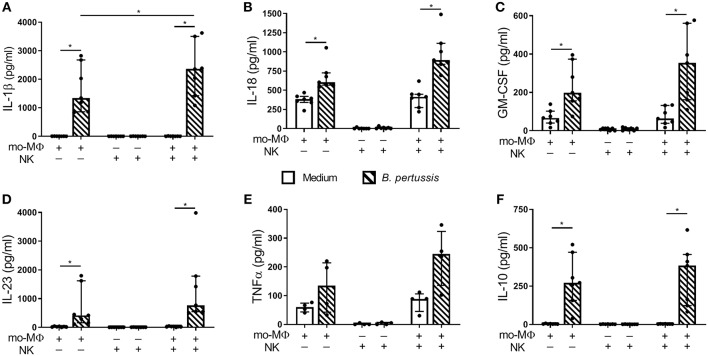
Increased proinflammatory cytokine secretion in *B. pertussis*-stimulated mo-MΦ/NK co-culture. Mo-MΦ and NK cell single cultures and mo-MΦ/NK co-cultures were stimulated with *B. pertussis* (B4393, MOI = 10, dashed bars) or left untreated (clear bars) for 22 h. Secreted levels of **(A)** IL-1β, **(B)** IL-18, **(C)** GM-CSF, **(D)** IL-23, **(E)** TNFα, and **(F)** IL-10 were measured in the supernatant (*n* = 7). Results are expressed as medians with interquartile range. Black dots represent values of individual donors. ^*^p < 0.05.

**Figure 5:**
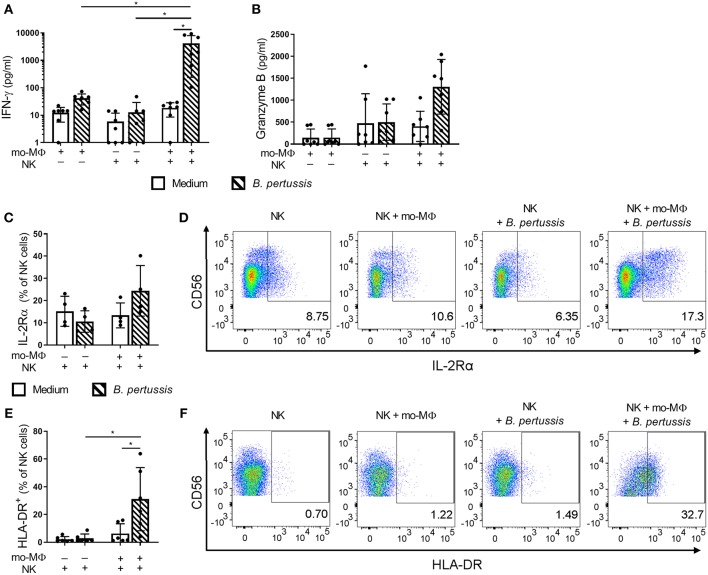
*B. pertussis*-stimulated mo-MΦ activate human NK cells. Mo-MΦ and NK cell single cultures and mo-MΦ/NK co-cultures were stimulated with *B. pertussis* (B4393, MOI = 10, dashed bars) or left untreated (clear bars) for 22 h. Secreted levels of **(A)** IFNγ and **(B)** Granzyme B were measured in the supernatant (*n* = 7). NK cells were stained for **(C,D)** IL-2Rα (*n* = 4) and **(E,F)** HLA-DR (*n* = 6) and the expression of these markers was analyzed on CD56^+^CD3^−^ NK cells. **(A–C,E)** Results are expressed as medians with interquartile range. Black dots represent values of individual donors. **(D,F)** Results are expressed as dot plots of one representative donor. ^*^p < 0.05.

To determine the potential effects of crosstalk between mo-MΦ and NK cells during *B. pertussis* stimulation, we characterized the cytokine profile of *B. pertussis*-stimulated mo-MΦ and NK cells cultured either separately or in co-culture. Stimulation of mo-MΦ with *B. pertussis* (B4393) resulted in a significant increase in the secretion of a wide range of cytokines, namely, IL-1β, IL-18, GM-CSF, IL-23, and IL-10 (Figures 4A–D,F). No significant increase in TNFα secretion was observed (Figure 4E). Interestingly, when mo-MΦ were stimulated with *B. pertussis* in the presence of equal amounts of autologous NK cells the secretion of all pro-inflammatory cytokines, IL-23, IL-1β, IL-18, GM-CSF, and TNFα increased (Figures 4A–E). The secretion of the anti-inflammatory cytokine IL-10 was not affected by the presence of NK cells (Figure 4F). These data indicate that, co-culture of NK cells and mo-MΦ induced a stronger pro-inflammatory cytokine response to *B. pertussis* compared to mo-MΦ stimulated alone. NK cells alone stimulated with *B. pertussis* did not result in the increased secretion of any of these cytokines compared to unstimulated NK cells (Figures 4A–F). However, in the *B. pertussis*-stimulated mo-MΦ/NK co-culture, secretion of the NK cell associated cytokine, IFNγ, was significantly increased compared to the *B. pertussis*-stimulated NK single culture (Figure 5A). Comparable findings on IFNγ secretion were observed when NK cells alone were stimulated with *B. pertussis* in the presence of rhIL-18 (Figure 3). Additionally, co-culture of mo-MΦ and NK cells resulted in an increased release of granzyme B (Figure 5B) as well as in the frequency of NK cells expressing IL-2Rα (Figures 5C,D) and HLA-DR (Figures 5E,F), indicating NK cell activation. These results show that there is crosstalk between mo-MΦ and NK cells in response to *B. pertussis*.

### IL-18 Contributes to NK Cell Activation and IFNγ Secretion in a *B. pertussis*-Stimulated mo-MΦ/NK Co-culture

**Figure 6:**
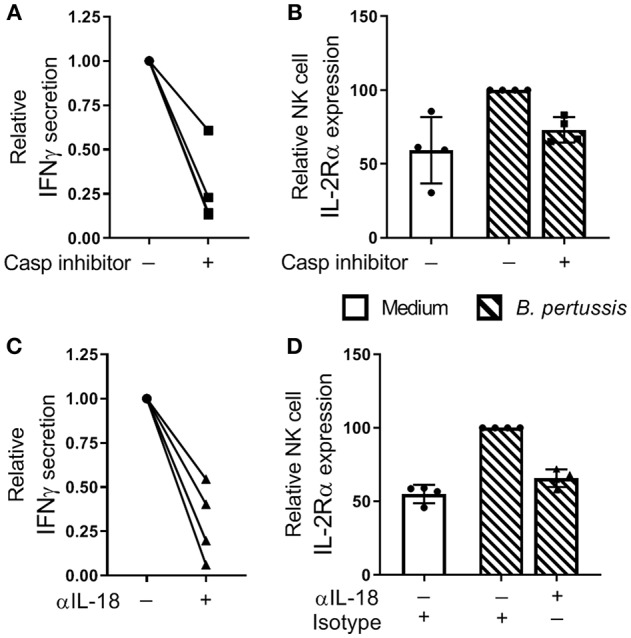
IL-18 contributes to NK cell activation and IFNγ secretion in a *B. pertussis*-stimulated mo-MΦ/NK co-culture. **(A,B)** Mo-MΦ/NK co-cultures were stimulated with *B. pertussis* (B4393, MOI = 10, dashed bars) for 22 h in the presence of a caspase inhibitor (squares). **(A)** IFNγ was measured in the supernatant and **(B)** IL-2Rα expression was determined on CD56^+^CD3^−^ NK cells using flow cytometry. Data is shown as relative to the cultures stimulated with *B. pertussis* in the absence of the caspase inhibitor (dots). **(C,D)** Mo-MΦ/NK co-cultures were stimulated with *B. pertussis* (B4393, MOI = 10, dashed bars) for 22 h in the presence of IL-18 blocking antibodies (triangles) or isotype control hIgA2 (dots). **(C)** IFNγ was measured in the supernatant and **(D)** IL-2Rα expression was determined on CD56^+^CD3^−^ NK cells using flow cytometry. Data is shown as relative to the cultures stimulated with *B. pertussis* in the presence of hIgA2 (*n* = 4). Results are expressed as medians with interquartile range. Black dots, squares and triangles represent values of individual donors.

To investigate the contribution of inflammasome activation in the *B. pertussis*-induced IFNγ secretion and NK cell activation, mo-MΦ/NK co-cultures were stimulated with *B. pertussis* (B4393) in the presence of a caspase inhibitor. This resulted in a reduction of 72.2% in IFNγ secretion relative to the cultures stimulated in the absence of a caspase inhibitor (Figure 6A). Additionally, the frequency of IL-2Rα^+^ NK cells was reduced by 27.8% (Figure 6B). To determine the role of IL-18 in the activation of NK cells and secretion of IFNγ, mo-MΦ/NK co-cultures were stimulated with *B. pertussis* in the presence of an IL-18 blocking antibody. This resulted in a reduction of 69.8% of the secreted IFNγ (Figure 6C) and a 34.2% reduction in the frequency of IL-2Rα^+^ NK cells (Figure 6D) whereas blocking IL-1β had no effect (Figure S4).

**Figure 7:**
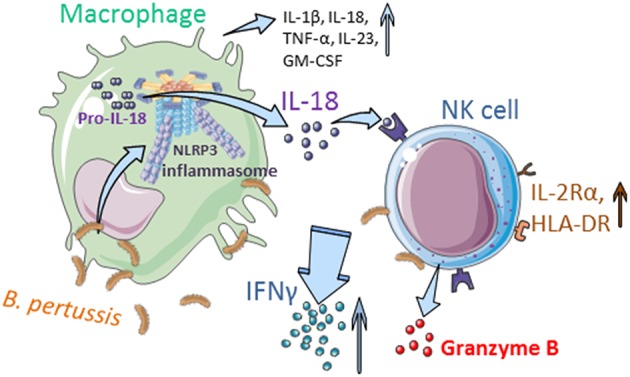
Interplay between human mo-MΦ and NK cells in the presence of *B. pertussis* (graphics). *B. pertussis* activates the NLRP3 inflammasome in human macrophages resulting in the secretion of, amongst others, IL-18 and IL-1β. IL-18 primes the NK cells to produce IFNγ and express IL-2Rα and HLA-DR in response to *B. pertussis*. Inflammasome activation and the crosstalk between human macrophages and NK cells results in an enhanced proinflammatory response to this pathogen (Made with illustrations from: https://smart.servier.com/).

Taken together, these data show that *B. pertussis* induces inflammasome activation in human macrophages resulting in IL-18 secretion, which is required for the activation of human NK cells and secretion of IFNγ upon encounter with this pathogen (Figure 7).

## Discussion

In this study, we show for the first time that *B. pertussis* induces activation of the NLRP3 inflammasome in human macrophages and that secreted IL-18 is required for NK cell activation by the pathogen. Furthermore, we show that the crosstalk between these innate cells in response to *B. pertussis* leads to an enhanced proinflammatory response.

Activation of the inflammasome complex is an early immune response involved in the induction of protective immunity against many different pathogens (30, 31). Here, we show using different approaches, that *B. pertussis* induces NLRP3 inflammasome formation in human macrophages resulting in caspase-mediated secretion of IL-1β and IL-18, as well as the induction of pyroptosis. In a study using murine DCs, *B. pertussis* was shown to induce activation of the NLRP3 inflammasome (9). The authors used modified variants of the adenylate cyclase toxin to show that this inflammasome activation was dependent on the pore forming ability of the adenylate cyclase toxin (9). Whether this virulence factor is also required for inflammasome activation in human innate cells remains to be determined. In another study in mice, Place et al. showed that IL-1β signaling is required for the clearance of *B. pertussis* and that the IL-1β is produced independent of caspase-1/caspase-11 (12). The authors suggest that an inflammasome-independent mechanism is involved in the *in vivo* secretion of IL-1β during a *B. pertussis* infection. In contrast to these findings, the same authors showed that *in vitro* the production of IL-1β by bone marrow-derived murine macrophages did require caspase-1. We show that in a human *in vitro* model, IL-1β production in response to *B. pertussis* involve both NLRP3 and caspase activity.

In addition to its role in innate immunity, inflammasome activation has been associated with the induction of a Th1/Th17 adaptive immune response (32–34), which are the protective type of T cell responses against *B. pertussis* (35–39). Using a murine infection model, IL-1β signaling was shown to be a critical step in promoting a protective Th17 response during *B. pertussis* infection (9) and to be essential in the clearance of *B. pertussis* (12). Whether inflammasome activation in human innate cells by *B. pertussis* contributes to polarization of the human adaptive response toward a Th1/Th17 phenotype remains to be investigated. In addition to IL-1β, we show that *B. pertussis-*stimulated human macrophages secrete IL-6 and IL-23, which are both associated with the induction of a Th17 response (40). These findings suggest that *B. pertussis*-stimulated human macrophages contribute to polarization toward a Th17 immune response.

A striking finding from our work is that the presence of IL-18 is required for activation of human NK cells and secretion of IFNγ in response to *B. pertussis*. IL-18 stimulation of human NK cells has been shown to stabilize IFNγ mRNA via the activation of mitogen-activated protein kinase p38 (27). This stabilization of IFNγ mRNA is a maturation step to prime NK cells for the production of IFNγ upon a secondary activation signal through stimulation of pattern recognition receptors (PRR) expressed on NK cells (41, 42). In accordance with this, Lauzon et al. showed that IL-2 treated human NK cells secreted IFNγ upon stimulation with TLR2, TLR3, TLR4, or TLR5 ligands (41). *B. pertussis* has been shown to activate different PRRs (43, 44), suggesting that PRRs stimulation provides the secondary activation signal required for IL-18-primed NK cells to produce IFNγ in response to *B. pertussis*.

Our results show that crosstalk between human macrophages and NK cells results in enhanced secretion of proinflammatory cytokines upon *B. pertussis* encounter. We highlight the critical role for inflammasome activity and IL-18 secretion in the induction of IFNγ secretion and NK cell activation in response to *B. pertussis*. Mahon et al. showed that IFNγ treatment of *B. pertussis*-infected murine macrophages resulted in reduced intracellular bacterial counts (23). Similarly, IFNγ has been shown to enhance the antimicrobial activity of human macrophages (45, 46) also against intracellular *B. pertussis* (47). Furthermore, studies in mice have shown the essential role for NK cells (20) and IFNγ (21, 23) in confining *B. pertussis* to the respiratory tract. Byrne et al. showed that *B. pertussis* infection of NK cell-depleted mice resulted in a lethal disseminating disease. Furthermore, the absence of NK cells resulted in reduced *B. pertussis*-specific IFNγ secretion and an increase in IL-5 secretion by spleen cells isolated 14 days after infection. This indicates a role for NK cells in skewing T cells toward a Th1 phenotype (20). Future studies should focus on the contribution of the cellular interplay between human macrophages and NK cells, or between other innate immune cells, in skewing T cell responses during *B. pertussis* infection. In addition to the increased secretion of pro-inflammatory cytokines in the *B. pertussis*-stimulated mo-MΦ/NK co-culture, we observed enhanced levels of the serine protease Granzyme B. This protease is released by cytotoxic cells, such as NK cells, and has been shown to kill bacteria such as *Escherichia coli, Listeria monocytogenes*, and *Mycobacteria tuberculosis* by cleaving electron transporters, oxidative stress defense proteins (48) and multiple proteins involved in protein synthesis as well as folding and degradation (49). Whether the Granzyme B in the *B. pertussis*-stimulated mo-MΦ/NK co-culture has a bactericidal effect on *B. pertussis* remains to be elucidated.

Taken together, our data provides a better understanding of the underlying mechanisms involved in the induction of innate immune responses against *B. pertussis*. Highlighted is the importance of the crosstalk between human macrophages and NK cells in enhancing proinflammatory responses against this pathogen and the role for inflammasome activation in this process. Knowledge on the mechanisms involved in the induction of protective immunity against *B. pertussis* is required for the development of improved intervention strategies to control this highly contagious disease.

## Data Availability

All datasets generated for this study are included in the manuscript and/or the Supplementary Files.

## Ethics Statement

This study was carried out in accordance with the recommendations of the Dutch Central Committee on Research involving human subjects with written informed consent from all subjects. All subjects gave written informed consent in accordance with the Declaration of Helsinki. The protocol required no review by an accredited Medical Research Ethics Committee, as determined by the Dutch Central Committee on Research involving human subjects.

## Author Contributions

MK, DH, H-JH, and KvB performed the experiments. MK drafted the figures. MK, RM, JvP, JdW, and EP contributed to the design of the study. MK, JvP, JdW, and EP wrote the manuscript. EP was responsible for funding. All authors approved the manuscript's final version.

### Conflict of Interest Statement

The authors declare that the research was conducted in the absence of any commercial or financial relationships that could be construed as a potential conflict of interest.
