# Supplementary material for: Activation of Human NK Cells by Bordetella pertussis Requires Inflammasome Activation in Macrophages
Source: Front Immunol. 2019 Aug 27;10:2030. doi: 10.3389/fimmu.2019.02030 (PMC6718514; doi:10.3389/fimmu.2019.02030)
Supplement: Supplementary file 1 [file Data_Sheet_1.docx]

Supplementary Material

# Supplementary Figures


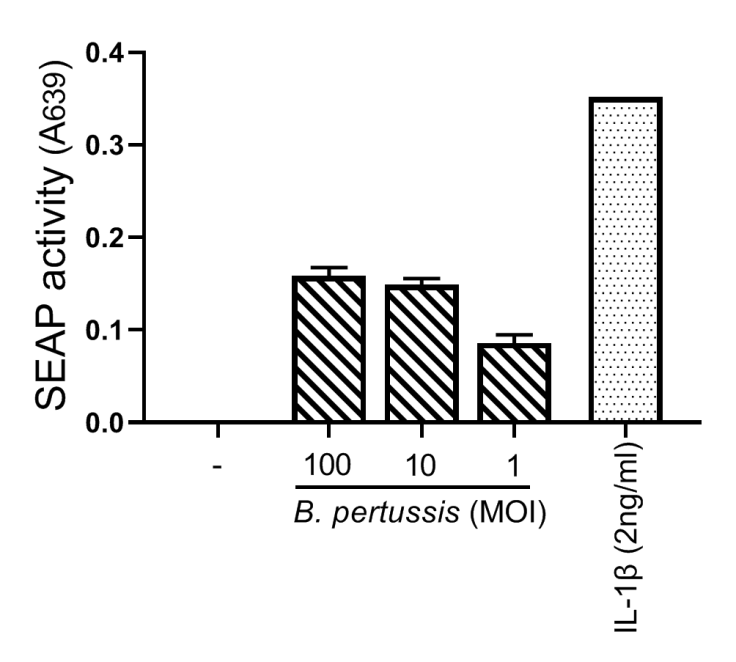


Figure S1. **B. pertussis *induces secretion of bioactive IL-1β by MΦ-like THP-1 cells*.** Supernatants from MΦ-like THP-1 cells that were left untreated (clear bars) or stimulated with *B. pertussis­* (B4393, MOI = 100, 10 or 1, dashed bar) were tested for bioactive IL-1β using the HEK-Blue IL-1β reporter cell line. As a positive control 2ng/ml IL-1β (dotted bar) was added. Results are expressed as SEAP activity indicating IL-1R signaling. Results are expressed as medians with interquartile range from five technical replicates.


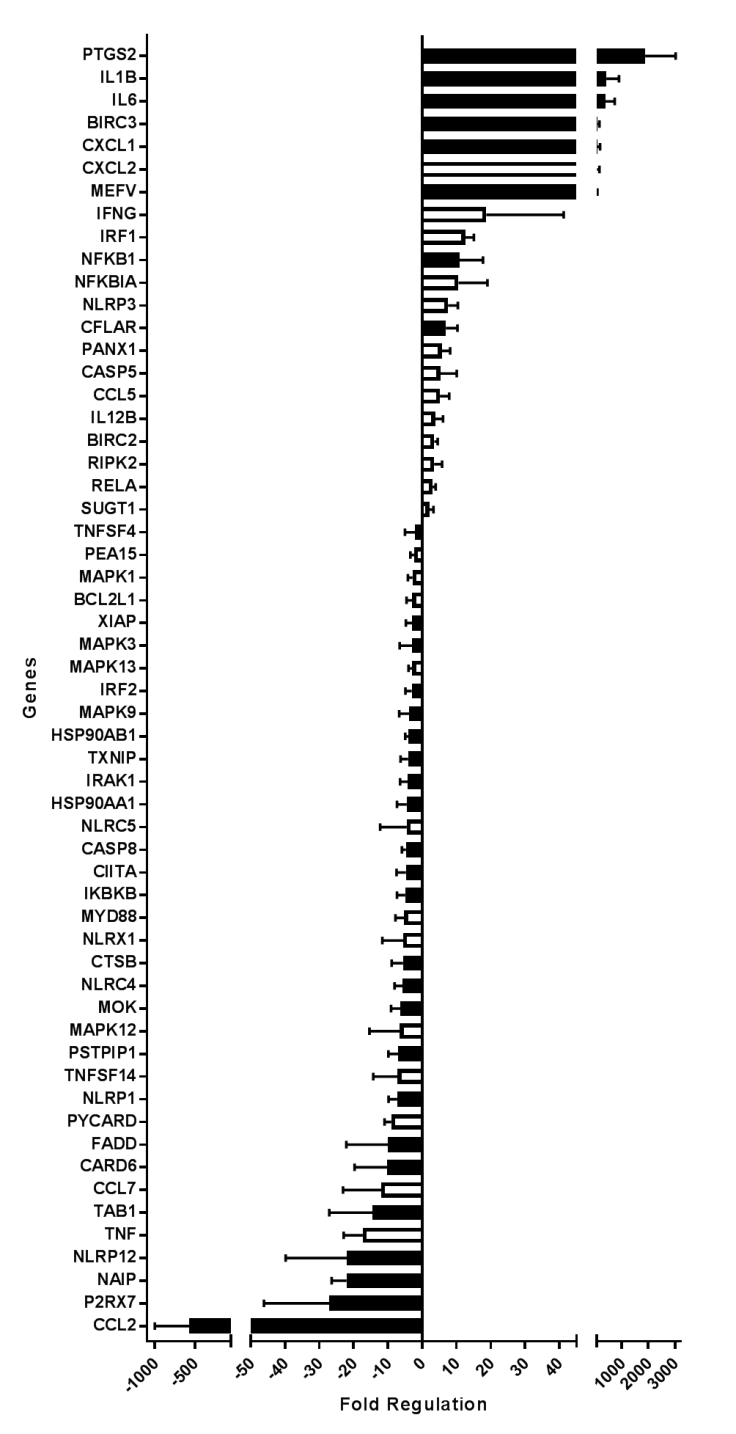


Figure S2. *Relative transcription levels of 84 inflammasome associated genes in* B. pertussis*-stimulated mo-MΦ***.** Genes with a fold regulation of >2 or <-2 of the transcription levels of mo-MΦ stimulated with *B. pertussis* (Tohama I, MOI = 100) for 6h relative to untreated mo-MΦ (n=3) measured with the QIAGEN human inflammasome qPCR Array. Black bars indicate statistically significant fold regulations (*p* = <0.05). Results are expressed as mean ± SD. Genes with a fold regulation between 2 and -2: *MAPK8*, *NOD1*, *NOD2*, *NLRP6*, *NFKBIB*, *IL12A*, *PYDC1*, *NLRP4*, *IL33*, *TNFSF11*, *IL18*, *MAPK11*, *TIRAP*, *TAB2*, *NLRP5*, *TRAF6*, *CD40LG*, *MAP3K7*, *NLRP9*, *AIM2*, *CARD18*, *IFNB1*, *BCL2*, *CHUK*, *IKBKG*, *CASP1*, *HSP90B1*.


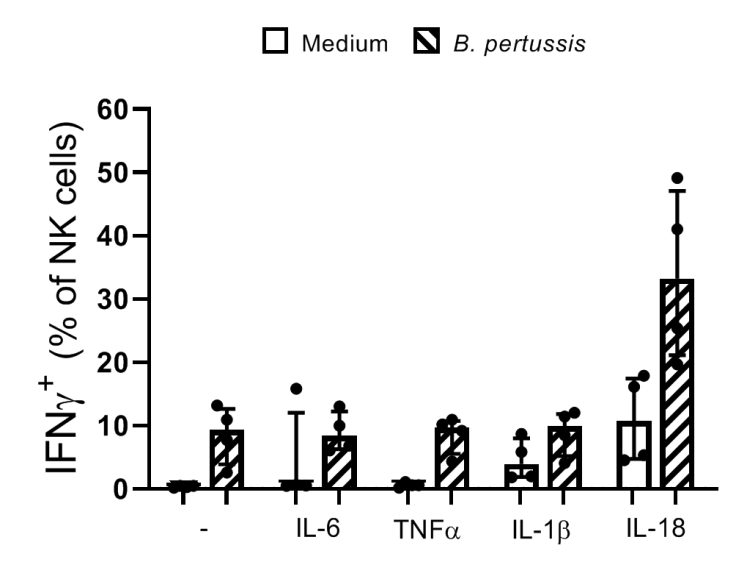


Figure S3. ***Specificity of IL-18 activation of NK cell IFNγ production*.** CD56^+^CD3^-^ NK cells were incubated with medium (clear bars) or *B. pertussis* (MOI = 10, B4393, dashed bars) in the presence or absence of 10ng/ml rhIL-6, rhTNFα, rhIL-1β or rhIL-18 for 18h after which Brefeldin A was added for 4h to inhibit cytokine secretion. Stimulated NK cells were intracellularly stained for IFNγ and the percentage of IFNγ^+^CD56^+^CD3^-^ NK cells was analyzed using flow cytometry (n=4). Results are expressed as medians with interquartile range. Black dots represent values of individual donors.


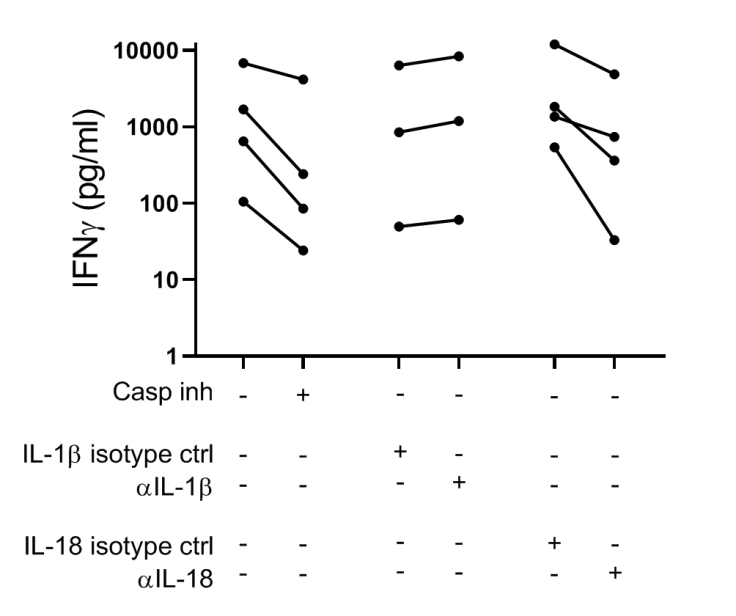


Figure S4. ***Specificity of the inflammasome in NK cell activation.*** Mo-MΦ/NK co-cultures were stimulated with *B. pertussis* (MOI = 10, B4393) in the presence or absence of a caspase inhibitor, blocking antibodies for IL-1β or IL-18 and their respective isotype controls. Secreted levels of IFNγ were measured in the supernatant (n=4).
